# Simulation of Mode I Interlaminar Damage of a GFRP Composite Using Cohesive Laws in the Framework of the Equivalent LEFM R-Curve and an Optimised Algorithm

**DOI:** 10.3390/polym13091482

**Published:** 2021-05-04

**Authors:** Luis Torres, Karin Saavedra, Gonzalo Pincheira, Juan Carlos Pina

**Affiliations:** 1 Magíster en Ciencias de la Ingeniería c/m Ingeniería Mecánica, Facultad de Ingeniería, Campus Curicó, Universidad de Talca, Curicó 3340000, Chile; ltorres12@alumnos.utalca.cl; 2 Departamento de Tecnologías Industriales, Campus Curicó, Universidad de Talca, Curicó 3340000, Chile; gpincheira@utalca.cl; 3 Departamento de Ingeniería en Obras Civiles, Facultad de Ingeniería, Universidad de Santiago de Chile (USACH), Santiago 9170124, Chile; juan.carlos.pina@usach.cl

**Keywords:** delamination, cohesive zone models, optimisation, linear elastic fracture mechanics

## Abstract

This paper is focused on mode I delimitation of a unidirectional glass fibre reinforced polymer (GFRP) composite. The aim is to propose an accurate and simple characterisation of three cohesive zone models (CZM)—bilinear, trilinear, and potential—from the measurement of the load-displacement curve during a double cantilever beam experimental test. For that, a framework based on the equivalent linear elastic fracture mechanics (LEFM) R-curve is here proposed, which has never before been developed for a bilinear and a potential CZM. Besides, in order to validate this strategy, an optimisation algorithm for solving an inverse problem is also implemented. It is shown that the parameters’ identification using the equivalent LEFM R-curve enables the same accuracy but reduces 72% the numerical efforts respect to a “blind fitting” (i.e., the optimisation algorithm). Therefore, even if optimisation techniques become popular at present due to their easy numerical implementation, strategies founded on physical models are still better solutions especially when evaluating the objective function is expensive as in mechanical problems.

## 1. Introduction

The use of structural composites in high performance applications, such as those present in the automotive or aerospace industry, has been steadily growing in during the last 50 years [1]. Despite the progress made on areas related to design, manufacturing and analysis of composites, one of the challenges that still remains is the accurate prediction of the progressive failure of the composite due to delamination [2]. These failure mechanisms are intrinsically related to the hierarchical nature of the laminates. Micro (ply), meso (laminate), and macro (structural) length scales should be considered to correctly predict the mechanical response of the composite. In this context, multi-scale modelling approaches that inherently incorporate the different scales present in composites, are a natural and frequently used alternative to study their mechanical response [3,4]. Furthermore, the increase in the use of numerical techniques, such as multi-scale modelling strategies, observed in the recent years, has led to a boost on the use of virtual testing techniques in the design and optimisation process of new laminate materials [5,6]. Additional benefits of virtual testing techniques are the cost and time reductions in the design process compared to experimental testing [6].

Failure mechanisms in composite laminates can be categorised as intraply (e.g., fibre fracture or matrix cracking) and interply (i.e., delamination) and, of course, complex interactions between them also can take place [7,8]. Moreover, it has been shown that the type of failure that is observed depends on the specimen size [9]. Delamination, defined as the crack propagation between adjacent plies, is one of the most relevant damage scenarios because it drastically reduces the mechanical strength and may lead to structural collapse [10,11,12,13]. The delamination fracture toughness is well established through the strain energy release rate $G_{i}$, according respectively to the three fracture modes ($G_{I}$, $G_{II}$ and $G_{III}$) and their combinations. The experimental procedures used to determine the delamination fracture toughness for each fracture mode are established in several standards [14,15,16,17]. Here, details on the specimens dimensions, loading protocol and data reduction are clearly outlined. The delamination occurs when the energy dissipated during fracture per unit of newly created surface is greater or equal than a critical strain energy release rate $G_{ic}$ (i.e., the resistance to crack growth), which can be viewed as a material property. Taking into account a general body with constant thickness *B* and an initial crack length $a_{0}$, under a loading *P* (N)ormal to the crack plane, the linear elastic fracture mechanics (LEFM) theory enables evaluating the fracture energy as follows [18]:

(1) $$G=\frac{P^{2}}{2B}\frac{dC(a)}{da}$$

where C(a)=δ/P is the compliance or displacement δ to applied load *P* ratio.

**Figure 1:**
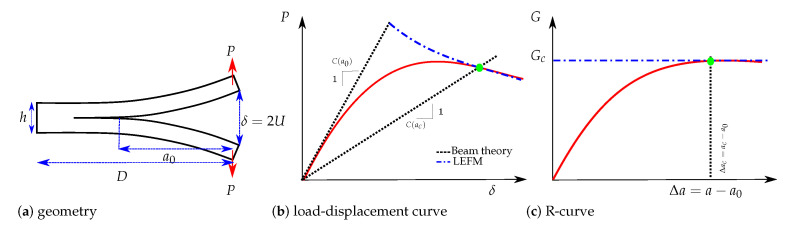
Double cantilever beam test.

The double cantilever beam (DCB) test shown in Figure 1a and used in this work is the most widely used procedure for the measurement of mode I delamination fracture toughness. For the load-displacement curve (see Figure 1b) and considering a rigid foundation at the crack ending, the compliance can be given by the beam theory as $C\left(a_{0}\right)=2a_{0}^{3}/3EI$, where $I=Bh^{3}/12$ and *E* is the Young’s module. A corrected compliance taking into account an elastic foundation is presented in [19]. Finally, the relation linking *P* and δ during the propagation phase can be obtained from Equation (1). Further details can be found in [20].

**Figure 2:**
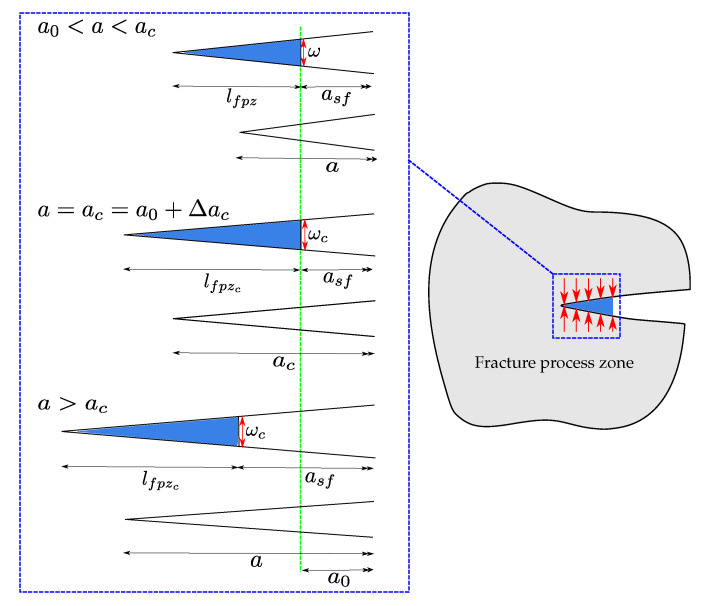
Fracture process zone and equivalent LEFM: $l_{fpz}$ is defined as the distance between the tip of the stress-free crack $a_{sf}$ and the point along the potential crack path where damage begins (schema inspired from [26]).

The R-curve shown in Figure 1c illustrates the variation of the material crack resistance with respect to the crack propagation length $\Delta a=a-a_{0}$. Ideal brittle materials present a flat R-curve, as shown by the blue line in Figure 1c. In this case, and when the crack propagates, the energy release rate $G_{I}$ remains constant and equal to $G_{Ic}$ (for the sake of simplicity subindex ${\left(.\right)}_{I}$ is omitted in the following). This behaviour is also observed when the crack propagation process of a material is studied by means of the LEFM method. Quasibrittle materials have a rising R-curve with an initiation phase where the resistance to the crack growth increases. Then the critical strain energy release rate reaches a steady-state plateau for a critical crack extension denoted as $\Delta a_{c}$, i.e., crack propagates in a self-similar steady way [21]. Rising R-curves require nonlinear fracture theories to describe the existence of a fracture process zone (FPZ) of length $l_{fpz}$ ahead of the crack tip (see Figure 2). The FPZ is where inelastic crack propagation mechanisms, such as fibre bridging and microcracks, take place. In fact, materials presenting large scale bridging have R-curves strongly dependent on the specimen’s geometry and therefore their constitutive damage model cannot be regarded as a material property [22,23]. More recently, the transition from 1D standard tests to 2D delamination scenarios has shown higher value of the fracture toughness for plates—due to stretching mechanisms affecting their stiffness—compared to the DCB specimens [24]. For all these quasibrittle behaviours, the R-curves need complex experimental setups for measuring the crack length, e.g., traveling microscope, crack gauge or video cameras. Another less expensive option is to use the equivalent LEFM [25], where the increase of the compliance can be related to the propagation of an equivalent LEFM crack. For any point of the experimental load-displacement curve in Figure 1b, a secant compliance is associated with each load; the corresponding equivalent crack extension is determined by solving the equation for C(a) and the crack growth resistance is then determined from Equation (1) [26].

**Figure 3:**
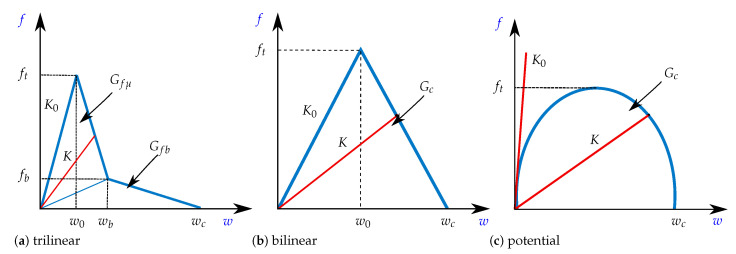
Traction-separation laws: (**a**) trilinear; (**b**) bilinear; and (**c**) potential CZM.

From a numerical point of view, there are a few techniques that can be used within a finite element (FE) method framework to address the delamination process in composites. Among the most frequently used strategies, there are adaptive remeshing procedures such as the virtual crack closure technique (VCCT) [27,28,29]; the use of enrichment functions near the crack tip such as the extended finite element method (X-FEM) [30,31,32,33]; or zero thickness interfaces with a continuum damage mechanics model such as the cohesive zone models (CZM) [34,35,36]. Advantages, limitations, and challenges of these three families of methods are discussed in [37]. If the crack path is known a priori, as in delamination of composite laminates, the CZM is the simplest and most accurate method. It has been widely used by researchers in recent decades for predicting both crack nucleation and propagation in composites [13,25,26,36,38,39,40,41,42,43,44,45,46,47,48,49,50,51]. The CZM method is defined by a constitutive law or softening function f(w) that relates the cohesive interface transfer traction *f* to the displacement jump *w*. The softening function is written in terms of un damage variable *d*, and characterised by a positive high initial stiffness $K_{0}$ and a maximum critical traction level $f_{t}$. Upon reaching $f_{t}$, the softening function is described by a negative tangent stiffness until a critical displacement jump $w_{c}$ is achieved. At this point, the system presents no more load-bearing capacity, i.e., $f(w_{c})=0$. As depicted in Figure 3, different forms of softening laws have been introduced such as linear, bilinear, trilinear, trapezoidal or exponential [52]. The area under the entire traction-displacement jump curve is the fracture energy $G_{c}$, i.e., the total energy required to completely separate the interface per unit area. These models are chosen according to a compromise between simple identification of its parameters and an accurate prediction of the crack propagation. In fact, due to the incapability to directly measure the f(w) curve, especially for materials with non-negligible FPZ, the model characterisation usually combines experimental data, theory (LEFM or *J*-Integral) or FE simulations. For example, it is possible to embed a fibre Bragg grating (FBG) sensor close to the crack tip and to measure the distributed strains [53,54,55], then an inverse method relates strains to the traction-separation curve. Digital image correlation (DIC) has also allowed inverse procedures combining full field cinematic data and FE simulations [56,57]. The approaches based on J-integral need experimental data such as the crack length, applied load or crack tip opening displacement (CTOD) [58,59,60,61]. In [62] a *J*-integral procedure is compared to an inverse optimisation scheme that minimises the difference between the experimental and simulated strains along the specimen. Although these techniques require very high resolution equipment to capture the CTOD or FPZ, which can cost a lot of money and be difficult to implement in specimens tested in a controlled environmental. Another option is to use inverse methods for minimising the residual between experimental and numerical P−δ curves [44,63,64,65,66]. However, despite their simplicity, they are very time-consuming because it is necessary several virtual tests to evaluate different values of each parameter of the softening function f(w). Moreover, FE simulations can be very expensive if models are more accurate, such as 6–8 CPU hours for only one 3D DCB [67]. In order to be more efficient, an inverse method combined with a model based on a Dugdale’s condition [68] or closed-form analytical solutions [69] has been developed for identifying multilinear, piecewise constant or bilinear CZM. Nevertheless, for more sophisticated shapes of CZM, optimisation algorithms seem to be the only possible way to identify the parameters. In this work, another alternative for reducing numerical and experimental efforts is exploited, which is based on the equivalent LEFM R-curve and has been initially proposed for a trilinear CZM [26]. To the best of the authors’ knowledge, this methodology has never before been developed for identifying a bilinear and a potential CZM or compared to “blind fitting” (i.e., an optimisation algorithm).

## 2. Experimental Test

DCB specimens were manufactured through a vacuum infusion process, considering a fibre/resin weight ratio of 0.47 and embedding a thin film at the mid-plane of the specimen for the pre-crack. Geometry (see Figure 1a) is specified in Table 1; an Epoxi 713 resin matrix with an E1174 hardener from the Chilean company Fibratec (Santiago, Chile) was employed, while the reinforcement is a unidirectional glass fibre from the German company P-D Interglas Technologies GmbH (now acquired by Porcher Industries, Eclose Badinieres, France), currently named as UD 220 g/m${}^{2}$ (i.e., with 207 g/m${}^{2}$ in the warp direction and 13 g/m${}^{2}$ in the fill direction). Then, the composite, previously characterised in [70], has the following elastic properties: $E_{1}=32.1$ GPa, $E_{2}=12.6$ GPa, $\nu_{12}=0.1$ (-), $\nu_{23}=0.24$ (-) and $G_{12}^{exp}=3$ GPa, where 1−direction is in the fibre direction and aligned through the specimen’s length.

**Figure 4:**
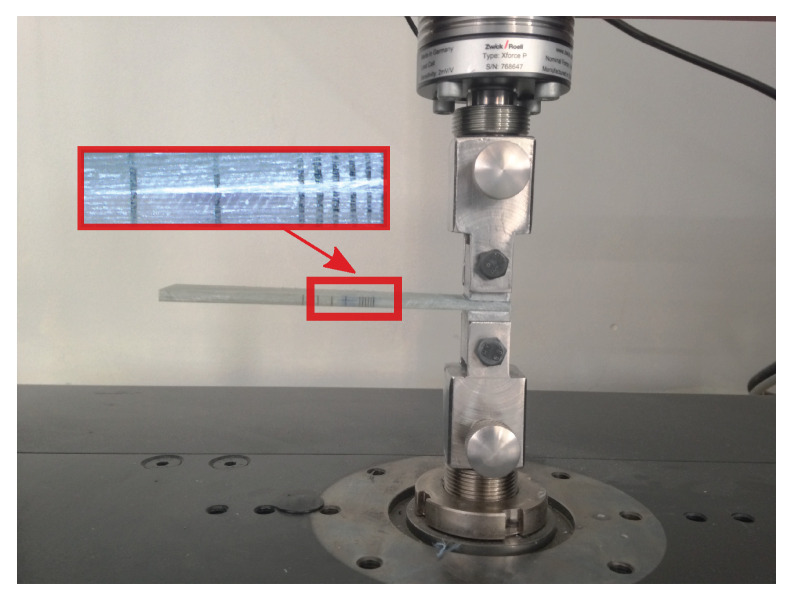
Experimental DCB tests: layout according to [17].

**Figure 5:**
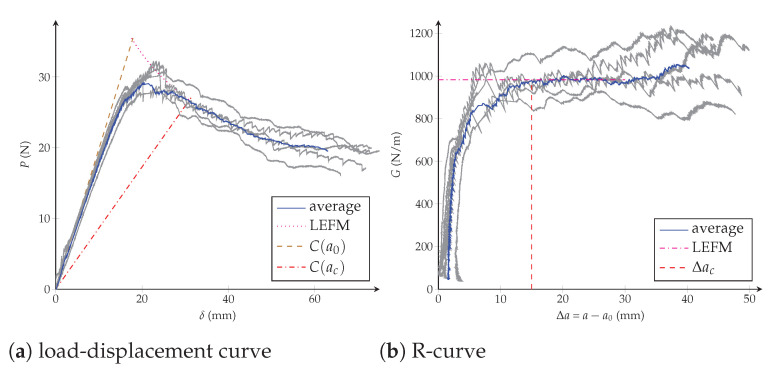
Experimental DCB tests: (**a**) load-displacement curve; (**b**) resistance curve.

The DCB test was carried out taking into account the ISO 15024 standard [17], under quasi-static conditions using a testing machine ZwickRoell (Ulm, Germany) provided with a 5 [kN] load cell and the testXpert testing software (see Figure 4). The load-displacement and resistance curves obtained from five experiments are drawn in Figure 5, whereas Table 2 summaries the critical energy release rate and the maximal applied load together with their standard deviations.

## 3. Cohesive Zone Models

Traction-separation laws f(w) can be written in terms of a damage variable *d*, ranging from 0 to 1, for a healthy to a completely damaged interface point, respectively. It is important to notice that in the tridimensional case f(w) is a vector-valued function, while the initial stiffness $K_{0}$ is a second order tensor. However, because this paper is only related to the mode I delamination, variables are all scalars. Therefore, the initial stiffness $K_{0}$ is progressively weaken according to:

(2) $$f\left(w\right)=\left(1-d\left({\langle w\rangle }_{+}\right)\right)K_{0}\;{\langle w\rangle }_{+}+K_{0}\;{\langle w\rangle }_{-}$$

where the symbols 〈〉 distinguish between the positive and negative part of the normal displacement in order to take into account the difference between tensile and compression. Considering the irreversibility of damage, *d* depends on the whole load history, i.e., $d_{{|}_{t}}=\max_{\tau \leq t}\left(d_{{|}_{\tau }}\right)$.

In this work, a bilinear [71,72], a trilinear [38,73] and a potential model [40] are studied, which are schematized in Figure 3. In all these laws, the fracture energy $G_{c}$ corresponds to the area under the curve. However, the energy is decomposed into two parts ($G_{c}=G_{f\mu }+G_{fb}$) for the trilinear CZM, which has been attributed to micro-cracking ($G_{f\mu }$) and fiber-bridging ($G_{fb}$) [73]. For each model, damage *d* is written in terms of the displacement jump *w* and the parameters to be identified, as summarized in Table 3.

## 4. CZM Characterization Using the Equivalent LEFM R-Curve

As explained in Section 1, the equivalent LEFM R-curve enables representing the influence of the FPZ development on the specimen compliance, through an elastically equivalent crack *a* (see Figure 2) located at some distance ahead of the initial crack $a_{0}$ (or the current stress-free crack $a_{sf}$). In [26], a new procedure to identify the parameters of the trilinear cohesive model has been proposed. It is based on the equivalent LEFM R-curve and on a dimensional analysis in order to relate the parameters’ dependency on the geometry and material of the specimen. The main idea is to relate each model parameter with the load-displacement curve and the corresponding equivalent LEFM R-curve through numerical simulations. Among the advantages, this methodology avoids an experimental measure of the critical opening $w_{c}$ and determines the CZM parameters more efficiently than blind fitting or optimization methods such as genetic algorithms, because less numerical evaluations are needed.

In this work, FE simulations of DCB tests are performed using 1D Euler-Bernoulli beam elements, coded through an OCTAVE routine, where only one half of the specimen—due to symmetry—is modelled and discretised into 310 finite elements. Geometry and elastic properties of the specimen are according to Section 2 (due to the 1D model, only $E_{1}=32.1$ GPa is taken into account), whereas the critical energy release rate is given by the experimental test ($G_{c}^{exp}=982.2$ N/m). In the following, the procedure defined for the trilinear CZM in [26] is applied to the fracture test of Section 3, then the methodology is extended to the bilinear and potential laws.

### 4.1. Trilinear CZM

The impact on the equivalent LEFM R-curve of each cohesive parameter is first analysed: the critical opening $w_{c}$, the distribution of the critical energy release rate $G_{f\mu }/G_{c}$ and the tensile strength $f_{t}$. The critical crack extension $\Delta a_{c}$ will be then associated with these parameters and the specimen size through a dimensional analysis. In this analysis $w_{0}=10^{-7}$ mm and $G_{c}=982.2$ N/m are kept constant.

**Figure 6:**
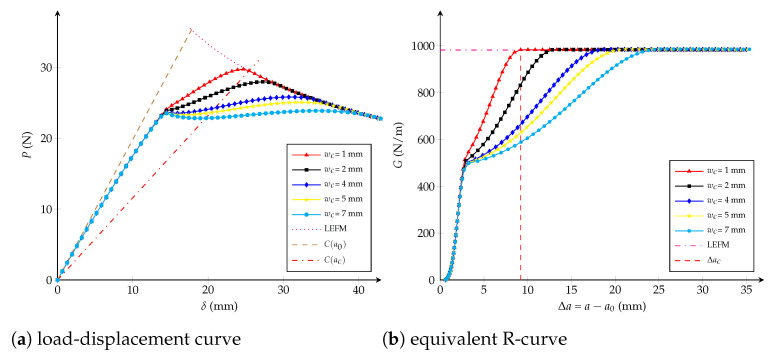
Trilinear cohesive model: (**a**) load-displacement curve; (**b**) equivalent R-curve. Influence of the critical opening $w_{c}$ with $f_{t}=1.15\cdot 10^{7}$ Pa and $G_{f\mu }/G_{c}=0.5$ (-) as constants.

**Figure 7:**
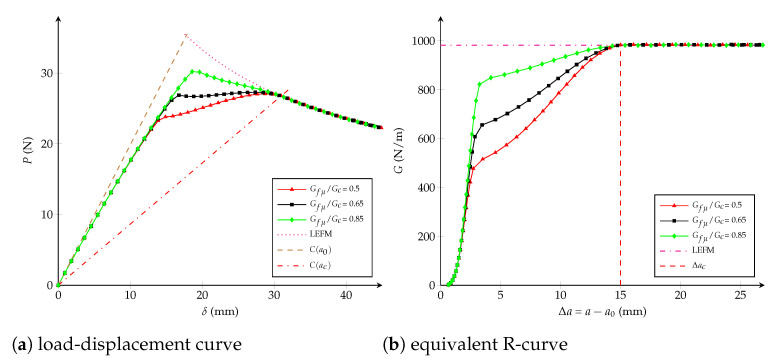
Trilinear cohesive model: (**a**) load-displacement curve; (**b**) equivalent R-curve. Influence of the fracture energy distribution $G_{f\mu }/G_{c}$ with $w_{c}=2.72$ mm and $f_{t}=$ 1.15$\cdot 10^{7}$ Pa as constants.

**Figure 8:**
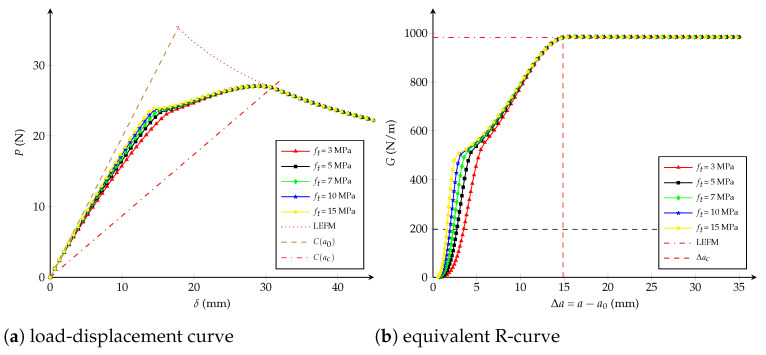
Trilinear cohesive model: (**a**) load-displacement curve; (**b**) equivalent R-curve. Influence of the tensile strength $f_{t}$ with $w_{c}=2.72$ mm and $G_{f\mu }/G_{c}=0.5$ (-) as constants.

- Influence of the critical opening: numerical simulations are carried out affecting the critical opening $w_{c}$∈{1,2,4,5,7} mm but fixing the values $G_{f\mu }/G_{c}=0.5$ (-) and $f_{t}=1.15\cdot 10^{7}$ Pa. From Figure 6, it is observed that $w_{c}$ does not have an influence before reaching the 50% of $G_{c}$. After that, the critical crack extension $\Delta a_{c}$ and the critical displacement jump $w_{c}$ have a positive correlation, which means that the length of the fracture process zone $l_{fpz}$ increases when $w_{c}$ increases.
- Influence of the fracture energy distribution: keeping constant $f_{t}=1.15\cdot 10^{7}$ Pa and $w_{c}=2.72$ mm, Figure 7 shows that at varying the critical energy release rate $G_{f\mu }/G_{c}\in \left\{0.5,0.65,0.75\right\}$ (-), the load-displacement plot and the equivalent LEFM R-curve are invariable if the dissipated energies $G_{f\mu }/G_{c}$ are lower than {0.5,0.65,0.75}, respectively. If the ratio $G_{f\mu }/G_{c}$ tends to one, the R-curve look like a one of a brittle material and the maximal load on the load-displacement curve increases. The critical crack extension $\Delta a_{c}$ always remains the same.
- Influence of the tensile strength: Figure 8 shows the impact at varying $f_{t}\in \left\{3,5,7,10,15\right\}$ [$10^{7}$ Pa] whereas $G_{f\mu }/G_{c}=0.5$ (-) and $w_{c}=2.72$ mm are unchanged. It can be concluded that the tensile strength impacts the response at the beginning of both curves: if $f_{t}$ increases, the behaviour becomes a brittle one. When the dissipated energy reaches the value $G_{f\mu }$, the fracture response starts to be the same for any value of $f_{t}$ and the critical crack extension $\Delta a_{c}$ becomes identical.

From the studies carried out in Figure 6, Figure 7 and Figure 8, the influence of each cohesive parameter when other variables are constant has been known. Now, from a dimensional analysis it can be supposed that $\Delta a_{c}$ depends on the specimen size and the cohesive parameters, as proposed in [26,74]:

(3) $$\frac{\Delta a_{c}}{l_{ch}}=\phi_{1}\left(\frac{D}{l_{ch}},\frac{w_{c}}{w_{ch}},\frac{G_{f\mu }}{G_{c}}\right)$$

where *D* is a characteristic dimension of the specimen, $l_{ch}$ and $w_{ch}$ are the Hillerborg’s characteristic length and a characteristic crack opening, respectively:

(4) $$l_{ch}=\frac{EG_{c}}{f_{t}^{2}}\;\;\;\;\;\;\;\;,\;\;\;\;\;\;\;\;w_{ch}=\frac{G_{c}}{f_{t}}$$

Because it was observed that the ratio $G_{f\mu }/G_{c}$ does not have influence on the critical crack extension $\Delta a_{c}$ (see Figure 7b), the third argument of Equation (3) is able to be directly vanished. According to Figure 8b, $\Delta a_{c}$ neither depends on $f_{t}$, therefore if Equation (3) is homogeneous in $f_{t}^{2}$—i.e., depends on $w_{c}^{2}$—it can be cancelled when factoring by $\frac{w_{c}^{2}}{w_{ch}^{2}}$ [26]:

(5) $$\frac{\Delta a_{c}}{l_{ch}}=\frac{w_{c}^{2}}{w_{ch}^{2}}\phi_{2}\left(\frac{Dw_{ch}^{2}}{w_{c}^{2}l_{ch}},1\right)\;\;\;\;\;\;\;\;\mathrm{or}\;\;\;\;\;\;\;\;\frac{\Delta a_{c}}{D}=\frac{w_{c}^{2}E}{G_{c}D}\phi \left(\frac{w_{c}^{2}E}{G_{c}D}\right)$$

where $\phi \left(\cdot \right)=\phi_{2}\left(1/\cdot ,1\right)$, furthermore the right equation has been multiplied by 1/D. On the other hand, a lower bound of the critical crack extension has been previously studied in [74] for D→∞, given by:

(6) $$\lim_{D\to \infty }\Delta a_{c}\approx \frac{\pi }{32}\frac{w_{c}^{2}E}{G_{c}}$$

Then, it is expected that ϕ(0)≈π/32.

The nonlinear expression relating $\Delta a_{c}$ and $w_{c}$, Equation (5), has the ability to be solved for $w_{c}$ through the equivalent LEFM R-curve and FE computations, according to:

(7) $$w_{c}=\sqrt{\frac{32\Delta a_{c}G_{c}}{\pi E}}\psi \left(\frac{\Delta a_{c}}{D}\right)$$

where ψ(0)≈1 when D→∞.

The critical opening $w_{c}$ can be now found employing Equation (7), while the other cohesive parameter—the tensile strength $f_{t}$—can also be studied through a dimensional analysis. Therefore, the crack length Δa, for a given energy release rate $G<G_{c}$, is allowed to be obtained from the following general expression:

(8) $$\frac{\Delta a}{l_{ch}}=\zeta_{1}\left(\frac{G}{G_{c}},\frac{D}{l_{ch}},\frac{w_{c}}{w_{ch}},\frac{G_{f\mu }}{G_{c}}\right)$$

From virtual tests previously carried out for different values of $w_{c}$ and $G_{f\mu }/G_{c}$, but keeping $f_{t}$ constant, it is observed that the equivalent R-curve remains almost the same when $G<0.5G_{c}$ (see Figure 6b and Figure 7b). In fact, $f_{t}$ only has an influence at the beginning of the R-curve if $w_{c}$ and $G_{f\mu }/G_{c}$ are both fixed (see Figure 8b). Therefore, function $\zeta_{1}\left(\cdot \right)$ is able to be considered independent of $w_{c}/w_{ch}$ and $G_{f\mu }/G_{c}$ if $G<0.5G_{c}$. For instance, calculations are here proposed with $G/G_{c}=0.2$ -:

(9) $$\frac{\Delta a_{0.2}}{l_{ch}}=\zeta_{2}\left(0.2,\frac{D}{l_{ch}}\right)=\zeta_{3}\left(\frac{D}{l_{ch}}\right)$$

**Figure 9:**
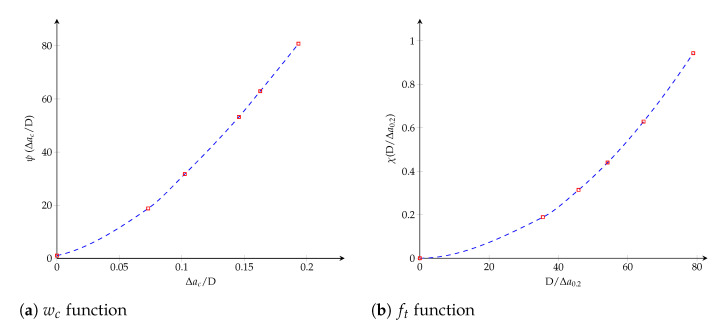
Trilinear cohesive model. The dimensionless functions for model parameters as a function of the crack length: (**a**) $w_{c}$ function; (**b**) $f_{t}$ function.

As proposed in [26], an expression relating $\Delta a_{0.2}/D$ and $D/l_{ch}$ is permitted to be obtained at multiplying Equation (9) by $l_{ch}/D$, then:

(10) $$f_{t}=\sqrt{\frac{EG_{c}}{D}}\chi \left(\frac{D}{\Delta a_{0.2}}\right)$$

where χ(·) can be determined from Figure 8b, considering the horizontal line for $G=0.2G_{c}$ and it verifies χ(0)≈0. Finally, the dimensionless functions ψ(·) and χ(·) plotted in Figure 9 are obtained through a Hermite spline cubic interpolation.

### 4.2. Bilinear CZM

The previous methodology is here developed for a bilinear model. In this case, it has been studied the influence of the critical opening $w_{c}$ and the initial stiffness $K_{0}$, while $G_{c}$ is always kept constant and equals to $G_{c}^{exp}$.

**Figure 10:**
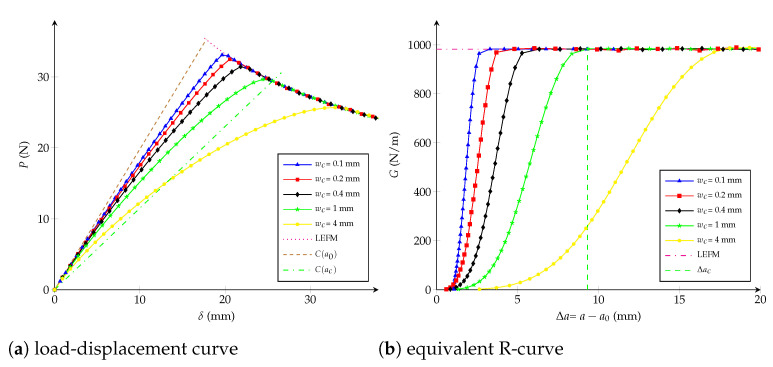
Bilinear cohesive model: (**a**) load-displacement curve; (**b**) equivalent R-curve. Influence of the critical opening $w_{c}$ with $K_{0}=10^{13}$ N/m${}^{3}$ as constant.

**Figure 11:**
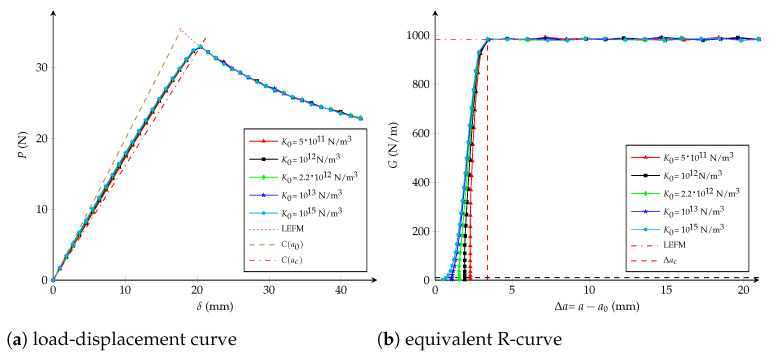
Bilinear cohesive model: (**a**) load-displacement curve; (**b**) equivalent R-curve. Influence of the initial stiffness $K_{0}$ with $w_{c}=0.13$ mm as constant.

- Influence of the critical opening: $w_{c}$ has an inverse correlation with the maximal applied load *P* when considering $w_{c}\in \left\{0.1,0.2,0.4,1,4\right\}$ mm and keeping unchanged $K_{0}=10^{13}$ N/m${}^{3}$, as observed in Figure 10a. Moreover, Figure 10b shows that the critical crack extension $\Delta a_{c}$ is inversely proportional to the tensile strength $f_{t}$, i.e., the interface becomes more brittle for higher values of $f_{t}$.
- Influence of the initial stiffness $K_{0}$: from Figure 11b it is observed that $K_{0}\in \left\{5,10,22,10^{2},10^{4}\right\}\cdot 10^{11}$ N/m${}^{3}$ does not have any influence on the critical crack extension $\Delta a_{c}$ if $f_{t}$ is kept constant. $K_{0}$ only has an effect on the beginning of the equivalent R-curve, which is not significant on the load-displacement curve (see Figure 11a).

As previously introduced in Section 4.1, the critical crack extension $\Delta a_{c}$ can be expressed as a function which depends on the specimen size and on the cohesive parameters as:

(11) $$\frac{\Delta a_{c}}{l_{ch}}=\gamma_{1}\left(\frac{D}{l_{ch}},\frac{w_{c}}{w_{ch}},\frac{K_{0}}{K_{ch}}\right)$$

where $K_{ch}$ is a characteristic stiffness defined as follows:

(12) $$K_{ch}=\frac{E^{4}}{G_{c}f_{t}^{2}}$$

From Figure 11b it is assumed that $K_{0}$ does not have influence on the critical crack extension $\Delta a_{c}$, then the third argument disappears. Besides, the relation $f_{t}=2G_{c}/w_{c}$ enables going without having the second variable $w_{c}/w_{ch}=0.5$, then multiplying by 1/D and considering Equation (4):

(13) $$\frac{\Delta a_{c}}{Dl_{ch}}=\frac{1}{D}\gamma_{2}\left(\frac{D}{l_{ch}}\right)\;\;\;\;\;\;\;\;\mathrm{or}\;\;\;\;\;\;\;\;\frac{\Delta a_{c}}{D}=\frac{w_{c}^{2}E}{4G_{c}D}\gamma_{2}\left(\frac{4G_{c}D}{w_{c}^{2}E}\right)\;\;\;\;\;\;\;\;\mathrm{or}\;\;\;\;\;\;\;\;\frac{\Delta a_{c}}{D}=\frac{w_{c}^{2}E}{G_{c}D}\gamma \left(\frac{w_{c}^{2}E}{G_{c}D}\right)$$

where it is expected, when D→∞, i.e., γ(0)≈π/32 in concordance with Equation (6). Finally, the critical opening is computed in the same way as for the trilinear law:

(14) $$w_{c}=\sqrt{\frac{32\Delta a_{c}G_{c}}{\pi E}}\eta \left(\frac{\Delta a_{c}}{D}\right)$$

where η(0)≈1 when D→∞.

To characterise the stiffness parameter $K_{0}$, a dimensionless general expression for the crack extension is able to be written as:

(15) $$\frac{\Delta a}{l_{ch}}=\nu_{1}\left(\frac{G}{G_{c}},\frac{D}{l_{ch}},\frac{w_{c}}{w_{ch}},\frac{K_{0}}{K_{ch}}\right)$$

From Figure 10b it should be noticed that the effect of $K_{0}$ on the R-curve depends on the selection of $w_{c}$. First, it is assumed that $w_{c}$ has been chosen using Equation (14); secondly, the crack extension is searched for a given *G* where the impact of $K_{0}$ is significative (e.g., $G/G_{c}=0.01$ -), subsequently last equation becomes:

(16) $$\frac{\Delta a_{0.01}}{l_{ch}}=\nu_{2}\left(\frac{D}{l_{ch}},\frac{K_{0}}{K_{ch}}\right)$$

The dependence on $f_{t}$ (i.e., on $w_{c}$) has the chance to be vanished if factoring by $\frac{E^{4}}{K_{0}G_{c}f_{t}^{2}}$, as follows:

(17) $$\frac{\Delta a_{0.01}}{l_{ch}}=\nu_{2}\left(\frac{Df_{t}^{2}}{EG_{c}},\frac{K_{0}G_{c}f_{t}^{2}}{E^{4}}\right)=\frac{K_{0}G_{c}f_{t}^{2}}{E^{4}}\nu_{3}\left(\frac{DE^{3}}{G_{c}^{2}K_{0}},1\right)$$

Then, multiplying last this expression by 1/D:

(18) $$\frac{\Delta a_{0.01}}{D}=\frac{K_{0}G_{c}^{2}}{DE^{3}}\nu_{3}\left(\frac{DE^{3}}{G_{c}^{2}K_{0}},1\right)=\frac{K_{0}G_{c}^{2}}{DE^{3}}\nu_{4}\left(\frac{DE^{3}}{G_{c}^{2}K_{0}}\right)=\nu \left(\frac{K_{0}G_{c}^{2}}{DE^{3}}\right)$$

**Figure 12:**
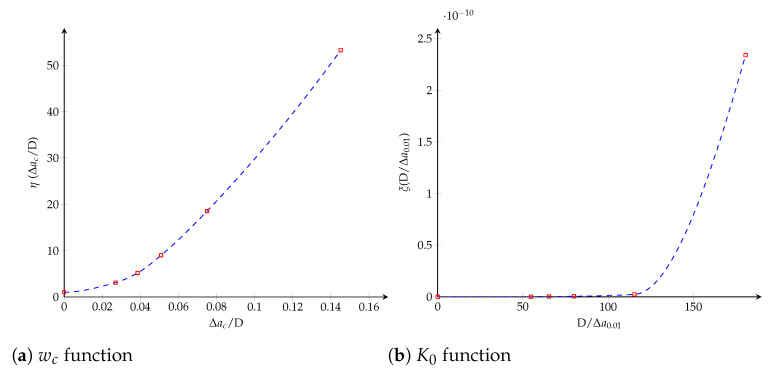
Bilinear cohesive model. The dimensionless functions for model parameters as a function of the crack length: (**a**) $w_{c}$ function; (**b**) $K_{0}$ function.

Finally, the initial stiffness can be stablished employing the next expression:

(19) $$K_{0}=\frac{DE^{3}}{G_{c}^{2}}\xi \left(\frac{D}{\Delta a_{0.01}}\right)$$

where it is verified that $K_{0}\left(\infty \right)\approx \infty$. The dimensionless functions, η(·) and ξ(·), characterizing both cohesive parameters are obtained through a Hermite spline cubic interpolation and plotted in Figure 12.

### 4.3. Potential CZM

**Figure 13:**
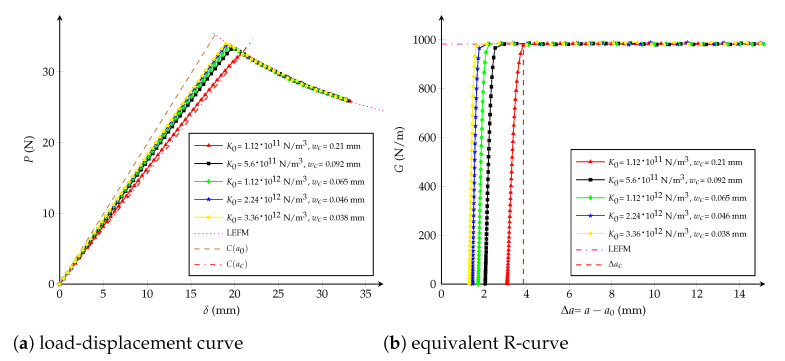
Potential cohesive model: (**a**) load-displacement curve; (**b**) equivalent R-curve. Influence of the initial stiffness $K_{0}$ with n=0.7 (-) as constant.

**Figure 14:**
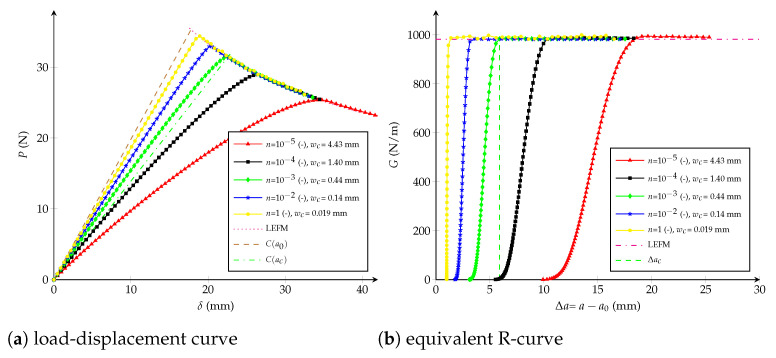
Potential cohesive model: (**a**) load-displacement curve; (**b**) equivalent R-curve. Influence of the parameter *n* with $K_{0}=1\cdot 10^{13}$ N/m${}^{3}$ as constant.

The parameters enabling identify the potential CZM are the initial stiffness $K_{0}$ and the dimensionless variable *n*. However, it is not possible to separately relate them to the critical crack extension $\Delta a_{c}$. Actually, for a fixed *n*, $K_{0}$ influences the whole R-curve (including $\Delta a_{c}$), as shown in Figure 13. Same behaviour takes place for different values of *n* but a fixed $K_{0}$, as demonstrated in Figure 14. In both cases $G_{c}$ is always kept constant and equals to $G_{c}^{exp}$.

Despite this impossibility, it is feasible to find a connection between (*n*, $K_{0}$) and $\Delta a_{c}$ if paying attention to the following relation from Table 3:

(20) $$d\left(w\right)={\left(\frac{\left(n+1\right)K_{0}w^{2}}{2nG_{c}}\right)}^{n}$$

More precisely, when considering d=1, the critical displacement jump is able to be written in terms of *n* and $K_{0}$ as follows:

(21) $$w_{c}^{2}=2G_{c}\frac{n+1}{nK_{0}}$$

Furthermore, it is possible to obtain an expression for $f_{t}$ through a stationary point of Equation (2), then:

(22) $$f_{t}=2^{-1/2n}\frac{nK_{0}}{{\left(n+\frac{1}{2}\right)}^{\frac{n+\frac{1}{2}}{n}}}{\left(2G_{c}\frac{n+1}{nK_{0}}\right)}^{1/2}$$

**Figure 15:**
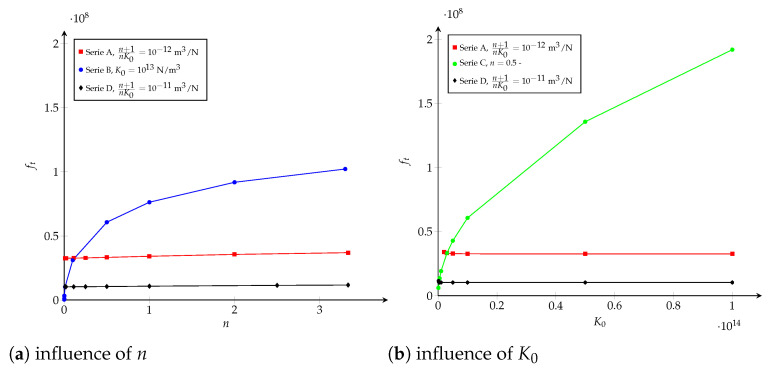
Potential cohesive model. Influence of (**a**) *n* and (**b**) $K_{0}$ on $f_{t}$ (more details in Table A1).

When examining Equation (22) for several values of *n* and $K_{0}$, as plotted in Figure 15 and detailed in Table A1 in Appendix A, it is noticed that $f_{t}$ is essentially unchanged if the term $\left(n+1\right)/\left(nK_{0}\right)$ (or $w_{c}$) remains invariable (series A and D). Nevertheless, if one parameter (*n* or $K_{0}$) is kept constant and the other is varied, $f_{t}$ increases when $K_{0}$ or *n* increases, respectively (series B and C). In the following, the effect of $\left(n+1\right)/\left(nK_{0}\right)$ on the equivalent R-curve is studied, while $G_{c}$ is equals to $G_{c}^{exp}$.

**Figure 16:**
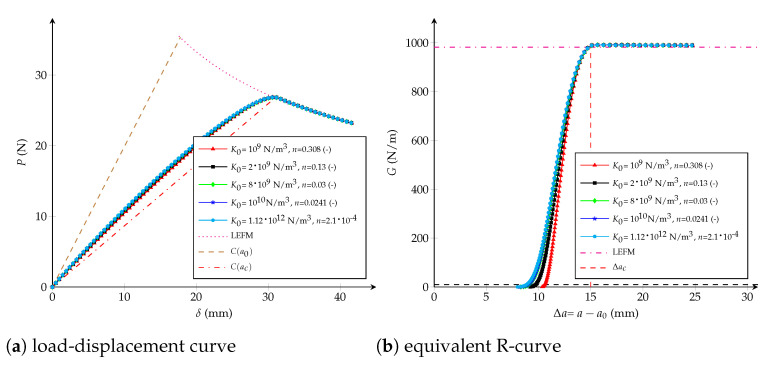
Potential cohesive model: (**a**) load-displacement curve; (**b**) equivalent R-curve. Influence of the cohesive parameters with $\frac{n+1}{nK_{0}}=4.25\cdot 10^{-9}$ m${}^{3}$/N ($w_{c}=2.9$ mm) as constant.

- Influence of $w_{c}$: considering n=0.7 (-) (respectively $K_{0}=10^{13}$ N/m${}^{3}$) unaltered, it is possible to observe the influence of $w_{c}$—or the influence of the term $\left(n+1\right)/\left(nK_{0}\right)$ according to Equation (21)—at varying $K_{0}\in \left\{1.12,5.6,11.2,22.4,33.6\right\}\cdot 10^{11}$ N/m${}^{3}$ (respectively $n\in \{10^{-5},10^{-4},10^{-3},10^{-2},10^{0}\}$ -). Figure 13b and Figure 14b show that the critical crack extension $\Delta a_{c}$ increases directly proportional to $w_{c}$.
- Influence of $K_{0}$ and *n*: when the critical opening is kept constant and equals to $w_{c}=2.9$ mm (or $\left(n+1\right)/\left(nK_{0}\right)=4.25\cdot 10^{-9}$ m${}^{3}$/N), but modifying $K_{0}\in \left\{2,8,10,1120\right\}\cdot 10^{9}$ N/m${}^{3}$ and $n\in \left\{1.13,0.3,3.8,0.24,0.0021\right\}\cdot 10^{-1}$ (-), the critical extension crack remains unchanged, as shown in Figure 16b. *n* and $K_{0}$ only have an influence in the beginning of the R-curve, while the load-displacement plot is almost the same.

With this previous analysis in mind, the general expression for the critical crack extension can be written as:

(23) $$\frac{\Delta a_{c}}{l_{ch}}=\mu_{1}\left(\frac{D}{l_{ch}},n,\frac{K_{0}}{K_{ch}}\right)$$

but it is allowed to be reformulated considering Equation (21) as follows:

(24) $$\frac{\Delta a_{c}}{l_{ch}}=\mu_{2}\left(\frac{D}{l_{ch}},\frac{w_{c}^{2}}{w_{ch}^{2}}\right)$$

Due to Figure 15b, let suppose that $f_{t}$ does not have an influence on $\Delta a_{c}$, then multiplying by 1/D Equation (24) turns into:

(25) $$\frac{\Delta a_{c}}{l_{ch}}=\frac{w_{c}^{2}}{w_{ch}^{2}}\mu_{3}\left(\frac{Dw_{ch}^{2}}{w_{c}^{2}l_{ch}},1\right)\;\;\;\;\;\;\;\;\mathrm{or}\;\;\;\;\;\;\;\;\frac{\Delta a_{c}}{D}=\frac{w_{c}^{2}E}{G_{c}D}\mu \left(\frac{Ew_{c}^{2}}{G_{c}D}\right)$$

which becomes identical to Equation (14) if solving for the critical crack opening:

(26) $$w_{c}=\sqrt{\frac{32\Delta a_{c}G_{c}}{\pi E}}\varsigma \left(\frac{\Delta a_{c}}{D}\right)$$

where ς(0)≈1 when D→∞.

Furthermore, the general expression for the crack extension, in terms of the potential CZM variables, is:

(27) $$\frac{\Delta a}{l_{ch}}=\beta \left(\frac{G}{G_{c}},\frac{D}{l_{ch}},n,\frac{K_{0}}{K_{ch}}\right)$$

Seeing that it is not possible to isolate and to directly choose $K_{0}$, it is then proposed to select $w_{c}$ at first and to vanish *n* in Equation (27)—because *n* depends on $K_{0}$ for a given $w_{c}$. Therefore, the impact of $K_{0}$ can be separated as observed in Figure 16b, especially in the first stage of the R-curve. Following that idea, the crack extension is then looked at a specific *G*, for example $G/G_{c}=0.01$ (-), by means:

(28) $$\frac{\Delta a_{0.01}}{l_{ch}}=\beta_{1}\left(\frac{D}{l_{ch}},\frac{K_{0}}{K_{ch}}\right)$$

**Figure 17:**
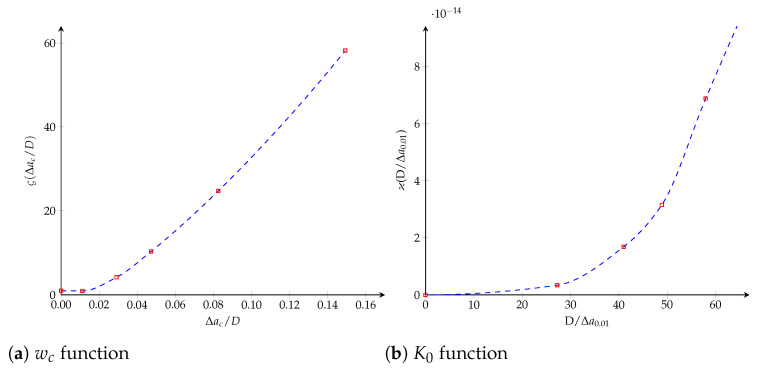
Potential cohesive model. The dimensionless functions for model parameters as a function of the crack length: (**a**) $w_{c}$ function; (**b**) $K_{0}$ function.

Because $f_{t}$ is supposed to be invariant when $w_{c}$ is fixed, the last relation becomes identical to Equation (16) and the initial stiffness is found in the same way as for the bilinear CZM:

(29) $$K_{0}=\frac{DE^{3}}{G_{c}^{2}}\varkappa \left(\frac{D}{\Delta a_{0.01}}\right)$$

where it is verified that $K_{0}\left(\infty \right)\approx \infty$. Finally, the dimensionless value *n* is established employing Equation (21). Functions ς(·) and ϰ(·), which are approached within Hermite spline cubic interpolations, are plotted in Figure 17.

## 5. Comparison of the CZM Characterization

**Figure 18:**
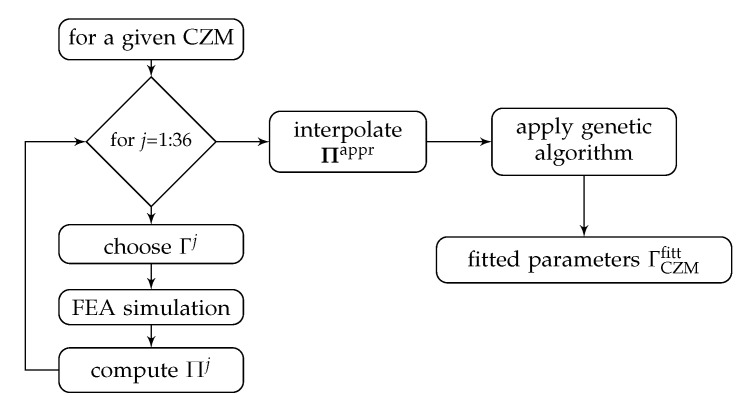
Schema of the optimization procedure.

The previously proposed methodology based on the equivalent LEFM R-curve is here applied to characterise the fibre-reinforced plastic tested in Section 2 under mode I fracture loading. The trilinear, bilinear and potential CZM are adjusted considering $G_{c}^{exp}\;=\;982.2$ N/m and $\Delta a_{c}^{exp}=15$ mm. At the same time, in order to verify the effectiveness of this characterization procedure, the parameters of these three laws are also looked for using an optimization algorithm. For this propose, an objective function Π is built from DCB simulations considering different sets of cohesive parameters $\Gamma^{j}$. More precisely, 36 sets of parameters $\Gamma^{j}$ are examined for each CZM, where each set enables finding one value of the objective function, considering the vertical difference between the experimental and numerical force-displacement curves, as follows:

(30) $$\Pi \left(\Gamma^{j}\right)=\Pi^{j}=\sum_{i=1}^{N}\frac{1}{N}{\left|P_{n,i}^{j}-P_{e,i}\right|}^{2}$$

where $P_{n,i}^{j}$ is a point in the numerical force-displacement curve considering the set of parameters $\Gamma^{j}$ whereas $P_{e,i}$ is the corresponding point in the experimental one. For each $\Pi^{j}$, the total number of points taken into consideration was N=20. From the 36 evaluations $\Pi^{j}$, the objective function Π is then approached through a spline interpolation leading to Π${}^{\mathrm{appr}}$—in order to avoid the expensive evaluation of Π—and finally it is minimized using a genetic algorithm, as detailed in Figure 18. For the latter, the Scilab optimization toolbox with settings in Table 4 is employed.

Table 5 summarizes the identified parameters $\Gamma_{\mathrm{CZM}}^{\mathrm{fitt}}$ for the three CZM considering both methodologies. To compare them, the value Π$\left(\Gamma_{\mathrm{CZM}}^{\mathrm{fitt}}\right)$ from Equation (30) is also computed. It is observed that the trilinear CZM achieves the best fitted parameters, for which the equivalent LEFM R-curve (Π = 0.33) is 28.3% lower than the genetic algorithm. In fact, the performance of the genetic algorithm is conditioned by the quality of Π${}^{\mathrm{appr}}$ which in turn depends on the amount of interpolated points $\Pi^{j}$, but these are expensive to obtain and thus are avoided. Furthermore, when applying the equivalent LEFM R-curve the bilinear and potential laws are not able to correctly emulate the experimental behaviour (Π= 16.2 and Π = 32.2, respectively). However, these both CZM are better adjusted with the genetic algorithm procedure (Π = 1.67 and Π = 2.22, respectively) but in any instance they are not more favourable than the trilinear law.

**Figure 19:**
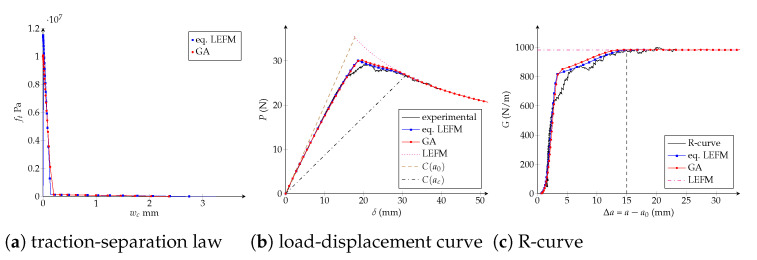
Trilinear cohesive model. Numerical DCB test with the fitted parameters: (**a**) traction-separation law; (**b**) load-displacement curve; (**c**) R-curve.

**Figure 20:**
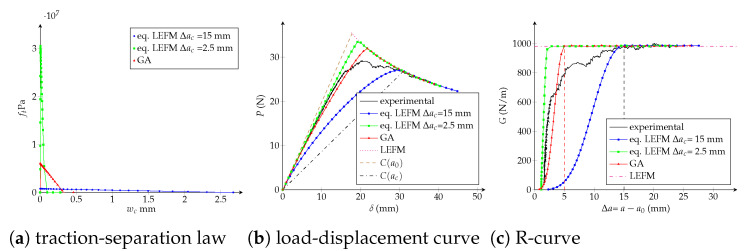
Bilinear cohesive model. Numerical DCB test with the fitted parameters: (**a**) traction-separation law; (**b**) load-displacement curve; (**c**) R-curve.

**Figure 21:**
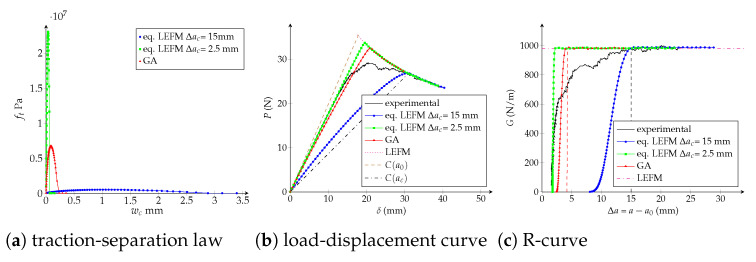
Potential cohesive model. Numerical DCB test with the fitted parameters: (**a**) traction-separation law; (**b**) load-displacement curve; (**c**) R-curve.

Traction-separation laws, load-displacement and R-curves are exposed in Figure 19, Figure 20 and Figure 21 for the trilinear, bilinear and potential models, respectively. It is confirmed the high-quality agreement of the trilinear CZM using both fitting methodologies, allowing reaching the critical crack extension $\Delta a_{c}$ as well as the maximal applied load *P* closely to the experimental values. The bilinear and potential laws are unable to follow the entire curves, indeed parameters obtained with the equivalent LEFM R-curve comply with the experimental critical crack extension but the initial response of the R-curve does not agree. On the other hand, parameters fitted with the genetic algorithm are able to follow the beginning of the load-displacement and R-curves; however, the critical crack extensions are lower than the empirical ones—33% (bilinear) and 29% (potential) lower than $\Delta a_{c}^{exp}$. Finally, and keeping in mind that the bilinear and potential laws are incapable of tuning behaviours with $\Delta a_{c}>>0$ (i.e., a quasibrittle material), it is reasonable to apply the equivalent LEFM R-curve method taking into account a flexible way to set $\Delta a_{c}$. For example, taking into account $\Delta a_{c}$ as the Δa when the fracture zone process starts to grow significantly in the R-curve. In Table 5 we include the model parameters considering $\Delta a_{0.56}$, subsequently a significant enhancement in the adjustment is achieved. In fact, the objective function is 80% (bilinear) and 91% (potential) lower than considering $\Delta a_{c}^{exp}$. Additionally, in Table 5 are listed the number of DCB virtual tests needed for each characterization methodology, a 72% of reduction is reached using the equivalent LEFM R-curve.

## 6. 3D Fracture Process Zone (FPZ)

**Figure 22:**
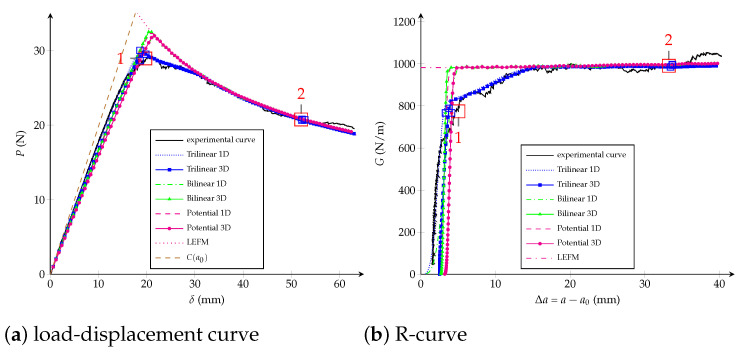
Experimental and numerical DCB tests (3D and 2D simulations): (**a**) load-displacement curve; (**b**) R-curve.

This section is devoted to studying the crack front of the DCB test considering the same geometry, material properties and boundary conditions which have been defined in Section 2. The goal is to perform 3D FE simulations and to contrast them against empirical observation, exploiting the fact that damage evolution can be directly observed because specimens are made of GFRP. Actually, in the experimental setup, a camera was placed perpendicularly to the crack plane for recording propagation from the top. FE simulations are carried out using a C++ research code called “MULTI” which is based on a parallel multiscale solver [75] and where the three CZM were implemented employing the best fitted parameters found for each law in Section 5 (see Table 5). The DCB sample is modelled using a 3D mesh with 365,552 linear tetrahedron elements and over two million degrees of freedom, the CZM is treated trough interfaces elements placed on the plane of delamination (78,744 2D triangular elements) between the upper and lower arms of the double cantilever beam. The load-displacement and R-curves are schematised in Figure 22, where 1D beam simulations are also included in order to verify that neglecting the orthotropic material properties (i.e., only $E_{1}=32.1$ GPa was considered in Section 4) was a proper assumption because the geometry and boundary conditions of the problem. Two instants are chosen to compare the plane of delamination: point 1 is located near to the maximum load carrying capacity and point 2 is placed faraway from the instant where propagation begun, as marked in Figure 22.

**Figure 23:**
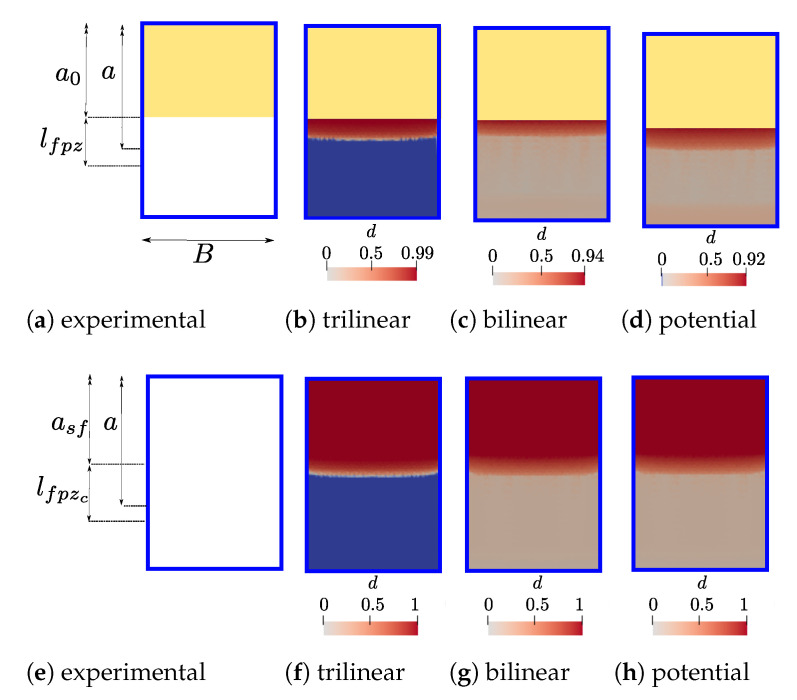
DCB delamination fronts for the experimental test and 3D simulations, according to points 1 (P1) and 2 (P2) from Figure 22: (**a**) experimental-P1; (**b**) trilinear-P1; (**c**) bilinear-P1; (**d**) potential-P1; (**e**) experimental-P2; (**f**) trilinear-P2; (**g**) bilinear-P2; (**h**) potential-P2. The initial pre-crack is indicated by a yellow area. The damage variable *d* ranges from 0 (blue) to 1 (red) in simulations.

Figure 23 presents a part of the delamination plane in order to show the crack tip for both instants of interest (points 1 and 2 in Figure 22). In order to monitor the crack growth, the experimental sample (see Figure 23a) is marked with 5 mm divisions along the delamination plane beyond of the tip of the pre-crack (that is the yellow area), but the first 5 mm are marked at 1 mm intervals. When the specimen is gradually loaded, it is possible to observe from the top view that the neighbourhood of the crack tip consistently turns a deep white. It is then supposed that this change in appearance is associated with the evolution of the FPZ and not only includes the crack propagation itself. The trilinear law has the thiner $l_{fpz}$ whereas the bilinear and potential have a very large $l_{fpz}$, which could be attributed to the initial stiffness $K_{0}$$\approx 10^{18}$ (trilinear), $\approx 10^{12}$ (bilinear) and $\approx 10^{11}$ (potential) N/m${}^{3}$, respectively for each law. Because the initial stiffness $K_{0}$ is also employed for the compression behaviour in simulations, low values can induce interpenetration and to enlarge the $l_{fpz}$. The damage distribution through the width is very similar in the four cases. Finally, it can be concluded that even if the load-displacement and resistence curves have a good agreement, the damage distribution over the length is not necessarily the same, especially for tiny values of *d*.

## 7. Conclusions

In this work, a new identification methodology for bilinear and potential CZM in mode I was developed, inspired by the strategy previously introduced by [26] for a trilinear cohesive law. The main idea is to relate each model parameter with the load-displacement curve and its corresponding equivalent LEFM R-curve through dimensional analyses and numerical simulations. This is implemented mainly in two steps: (1) obtaining the experimental load-displacement test on DCB specimens, computation of the equivalent LEFM R-curve, the critical strain energy release rate $G_{c}$ and the critical crack extension $\Delta a_{c}$; (2) computation of the critical opening $w_{c}$ and the other corresponding model parameters from dimensionless functions depending on geometry and material of the specimen. Please note that the order of the data reduction for each parameter is crucial. Among the advantages, the parameters’ identification based on the equivalent LEFM R-curve only needs the experimental load-displacement curves, avoiding sophisticated experimental setups and thus it drastically reduces the costs. For validating this strategy, an optimisation algorithm for solving an inverse problem is also here implemented for comparing the identification of bilinear, trilinear and potential laws. Then, the following conclusions are drawn as a result of this research:

- it is possible to characterise a bilinear and a potential CZM using a framework based on the equivalent LEFM R-curve;
- for the linear, bilinear and potential CZM, the parameters’ identification based on the equivalent LEFM R-curve enables the same accuracy but reduces 72% the numerical efforts respect to a “blind fitting” which minimise the residual between experimental and numerical load-displacement curves;
- when applying the equivalent LEFM R-curve framework for characterising a quasibrittle GFRP, the trilinear law achieves the best adjustment which is also proven comparing 3D simulations of the fracture process zones. However, it is expected that a trilinear CZM fits materials with large FPZ better than bilinear and potential models. Latter will be fully exploited when characterising more brittle materials;
- finally, even if optimisation techniques become popular at present due to their easy numerical implementation, strategies founded on physical models are still better solutions especially when evaluating the objective function is expensive as in mechanical problems.

## Figures and Tables

**Table 1:** DCB specimen geometry according to [17].

Thickness *h* (mm)	Width *B* (mm)	Length *D* (mm)	Pre-Crack $a_{0}$ (mm)
2.72 ± 0.07	20.4 ± 0.08	124.68 ± 0.52	47

**Table 2:** Experimental DCB tests: critical energy release rate and maximal applied load.

Specimen	$G_{c}^{\exp}$ (N/m)	$P_{\max}^{\exp}$ (N)
1	981.65	31.08
2	926.60	32.16
3	1062.80	29.72
4	964.90	27.98
5	974.8	30.70
average value	982.15	30.33
standard deviation	49.85	1.58

**Table 3:** Damage functions d(w) and parameters of the bilinear, trilinear and potential CZM.

CZM	Trilinear [26]	Bilinear [72]	Potential [40]
	d=0, $\;\;\;w<w_{0}$	d=0, $\;\;\;w\leq w_{0}$	$d=min\left({\left(\frac{n}{n+1}\frac{Y}{G_{c}}\right)}^{n},1\right)$
	$d=\frac{w_{b}\left(w-w_{o}\right)\left(1-\gamma \right)}{w(w_{b}-w_{o})},\;\;\;w_{o}\leq w\leq w_{b}$	$d=\frac{w_{c}}{w_{c}-w_{0}}\left(\frac{w-w_{0}}{w}\right)$, $\;\;\;w_{0}<w\leq w_{c}$	where $Y=\frac{1}{2}K_{0}w^{2}$
damage law	$d=1-\frac{\gamma w_{b}\left(w_{c}-w\right)}{w(w_{c}-w_{b})},\;\;\;w_{b}\leq w\leq w_{c}$	d=1, $w>w_{c}$	
	where $\gamma =\frac{f_{b}w_{o}}{f_{t}w_{b}},\;\;\;w_{0}=\frac{f_{t}}{K_{0}},$	where $w_{0}=\frac{f_{t}}{K_{0}},\;\;\;f_{t}=\frac{2G_{c}}{w_{c}}$	
	$w_{b}=\frac{2G_{f\mu }}{ft},\;\;\;f_{b}=\frac{2G_{fb}}{w_{c}}$		
parametersto be identified	$w_{c}$, $G_{f\mu }/G_{c}$, $f_{t}$	$w_{c}$, $K_{0}$	$K_{0}$, *n*

**Table 4:** Parameters for the genetic algorithm.

poblation size	5000
crossover probability	0.7
mutation probability	0.1
number of generation	300
number of couples	500
pressure	0.05

**Table 5:** Fitted parameters for each cohesive model using equivalent LEFM and genetic algorithm.

CZM	Characterization	Number of DCB Virtual Tests	Π $\left(\Gamma_{\mathrm{CZM}}^{\mathrm{fitt}}\right)$	$\Delta a_{c}$ (mm)	Fitted Parameters $\Gamma_{\mathrm{CZM}}^{\mathrm{fitt}}$
trilinear	eq. LEFM R-curve	10	0.33	15.0	$f_{t}=11.51$ MPa
					$w_{c}=2.71$ mm
					$G_{fu}/G_{c}=0.83$ (-)
	genetic algorithm	36	0.46	14.5	$f_{t}=10.08$ MPa
					$w_{c}=2.27$ mm
					$G_{fu}/G_{c}=0.85$ (-)
bilinear	eq. LEFM R-curve	10	16.2	15.0	$w_{c}=2.62$ mm
					$K_{0}=2.45\cdot 10^{12}$ N/m${}^{3}$
	eq. LEFM R-curve	10	3.21	2.5	$w_{c}=0.064$ mm
					$K_{0}=8.67\cdot 10^{12}$ N/m${}^{3}$
	genetic algorithm	36	1.67	4.96	$w_{c}=0.32$ mm
					$K_{0}=1.151\cdot 10^{12}$ N/m${}^{3}$
potential	eq. LEFM R-curve	10	32.2	15.0	$n=2.1\cdot 10^{-4}$ (-)
					$K_{0}=1.12\cdot 10^{12}$ N/m${}^{3}$
	eq. LEFM R-curve	10	2.98	2.5	n=0.86 (-)
					$K_{0}=1\cdot 10^{12}$ N/m${}^{3}$
	genetic algorithm	36	2.22	4.3	n=0.1 (-)
					$K_{0}=4.71\cdot 10^{11}$ N/m${}^{3}$

## Data Availability

Data is contained within the article.

## Appendix A

Table A1 summaries the study carried out to understand the influence of the cohesive parameters on the critical traction $f_{t}$ and on the critical displacement jump $w_{c}$ in the case of the potential law.

**Table A1:** Potential cohesive model. Influence of model parameters on $f_{t}$ and $w_{c}$.

	$K_{0}$ (N/m${}^{3}$)	n (-)	$\frac{n+1}{{\mathrm{nK}}_{0}}$ (m${}^{3}$/N)	$w_{c}$ (m)	$f_{t}$ (Pa)
	1 $\cdot 10^{14}$	0.01	1 $\cdot 10^{-12}$	4.43 $\cdot 10^{-5}$	3.26 $\cdot 10^{7}$
	5 $\cdot 10^{13}$	0.02	1 $\cdot 10^{-12}$	4.43 $\cdot 10^{-5}$	3.26 $\cdot 10^{7}$
	1 $\cdot 10^{13}$	0.11	1 $\cdot 10^{-12}$	4.43 $\cdot 10^{-5}$	3.27 $\cdot 10^{7}$
serie A	5 $\cdot 10^{12}$	0.25	1 $\cdot 10^{-12}$	4.43 $\cdot 10^{-5}$	3.28 $\cdot 10^{7}$
	3 $\cdot 10^{12}$	0.5	1 $\cdot 10^{-12}$	4.43 $\cdot 10^{-5}$	3.32 $\cdot 10^{7}$
	2 $\cdot 10^{12}$	1	1 $\cdot 10^{-12}$	4.43 $\cdot 10^{-5}$	3.41 $\cdot 10^{7}$
	1.5$\cdot 10^{12}$	2	1 $\cdot 10^{-12}$	4.43 $\cdot 10^{-5}$	3.56 $\cdot 10^{7}$
	1.3$\cdot 10^{12}$	3.3	1 $\cdot 10^{-12}$	4.43 $\cdot 10^{-5}$	3.69 $\cdot 10^{7}$
	1 $\cdot 10^{13}$	2	1.5$\cdot 10^{-13}$	1.72 $\cdot 10^{-5}$	9.18 $\cdot 10^{7}$
	1 $\cdot 10^{13}$	1	2$\cdot 10^{-13}$	1.98 $\cdot 10^{-5}$	7.63 $\cdot 10^{7}$
	1 $\cdot 10^{13}$	0.5	3$\cdot 10^{-13}$	2.43 $\cdot 10^{-5}$	6.07 $\cdot 10^{7}$
serie B	1 $\cdot 10^{13}$	0.1	1.1$\cdot 10^{-12}$	4.65 $\cdot 10^{-5}$	3.11 $\cdot 10^{7}$
	1 $\cdot 10^{13}$	0.01	1$\cdot 10^{-11}$	1.41 $\cdot 10^{-5}$	1.03 $\cdot 10^{7}$
	1 $\cdot 10^{13}$	0.001	1$\cdot 10^{-10}$	4.43 $\cdot 10^{-4}$	3.26 $\cdot 10^{6}$
	1 $\cdot 10^{13}$	0.0001	1$\cdot 10^{-9}$	1.4 $\cdot 10^{-3}$	1.03 $\cdot 10^{6}$
	1 $\cdot 10^{13}$	0.00001	1$\cdot 10^{-8}$	4.43 $\cdot 10^{-3}$	3.26 $\cdot 10^{5}$
	1 $\cdot 10^{14}$	0.5	3 $\cdot 10^{-14}$	7.68 $\cdot 10^{-6}$	1.92 $\cdot 10^{8}$
	5 $\cdot 10^{13}$	0.5	6 $\cdot 10^{-14}$	1.09 $\cdot 10^{-5}$	1.36 $\cdot 10^{8}$
	1 $\cdot 10^{13}$	0.5	3 $\cdot 10^{-13}$	2.43 $\cdot 10^{-5}$	6.07 $\cdot 10^{7}$
serie C	5 $\cdot 10^{12}$	0.5	6 $\cdot 10^{-13}$	3.43 $\cdot 10^{-5}$	4.29 $\cdot 10^{7}$
	3 $\cdot 10^{12}$	0.5	1 $\cdot 10^{-12}$	4.43 $\cdot 10^{-5}$	3.32 $\cdot 10^{7}$
	1 $\cdot 10^{12}$	0.5	3 $\cdot 10^{-12}$	7.68 $\cdot 10^{-5}$	1.92 $\cdot 10^{7}$
	5 $\cdot 10^{11}$	0.5	6 $\cdot 10^{-12}$	1.09 $\cdot 10^{-4}$	1.36 $\cdot 10^{7}$
	1 $\cdot 10^{11}$	0.5	3 $\cdot 10^{-11}$	2.43 $\cdot 10^{-4}$	6.07 $\cdot 10^{6}$
	1 $\cdot 10^{14}$	0.001	1 $\cdot 10^{-11}$	1.40 $\cdot 10^{-4}$	1.03 $\cdot 10^{7}$
	5 $\cdot 10^{13}$	0.002	1 $\cdot 10^{-11}$	1.40 $\cdot 10^{-4}$	1.03 $\cdot 10^{7}$
	1 $\cdot 10^{13}$	0.0101	1 $\cdot 10^{-11}$	1.40 $\cdot 10^{-4}$	1.03 $\cdot 10^{7}$
serie D	5 $\cdot 10^{12}$	0.0204	1 $\cdot 10^{-11}$	1.40 $\cdot 10^{-4}$	1.03 $\cdot 10^{7}$
	1 $\cdot 10^{12}$	0.111	1 $\cdot 10^{-11}$	1.40 $\cdot 10^{-4}$	1.03 $\cdot 10^{7}$
	5 $\cdot 10^{11}$	0.25	1 $\cdot 10^{-11}$	1.40 $\cdot 10^{-4}$	1.04 $\cdot 10^{7}$
	3 $\cdot 10^{11}$	0.5	1 $\cdot 10^{-11}$	1.40 $\cdot 10^{-4}$	1.05 $\cdot 10^{7}$
	2 $\cdot 10^{11}$	1	1 $\cdot 10^{-11}$	1.40 $\cdot 10^{-4}$	1.08 $\cdot 10^{7}$
